# Trends in molecular characteristics and antimicrobial resistance of group B streptococci: a multicenter study in Serbia, 2015–2020

**DOI:** 10.1038/s41598-020-79354-3

**Published:** 2021-01-12

**Authors:** Dusan Kekic, Ina Gajic, Natasa Opavski, Milan Kojic, Goran Vukotic, Aleksandra Smitran, Lidija Boskovic, Marina Stojkovic, Lazar Ranin

**Affiliations:** 1 Institute for Microbiology and Immunology, Medical Faculty, University of Belgrade, Dr Subotica No. 1, 11000 Belgrade, Serbia; 2 Institute of Molecular Genetics and Genetic Engineering, University of Belgrade, 11000 Belgrade, Serbia; 3 Faculty of Biology, University of Belgrade, 11000 Belgrade, Serbia; 4 Institute for Microbiology and Immunology, Medical Faculty, University of Banja Luka, 78000 Banja Luka, Bosnia and Herzegovina; 5 Department for Microbiology, University Hospital Center “Dr Dragisa Misovic”, 11000 Belgrade, Serbia; 6 Department for Microbiology, The Obstetrics and Gynaecology Clinic “Narodni Front”, 11000 Belgrade, Serbia

**Keywords:** Microbiology, Molecular biology, Health care, Molecular medicine

## Abstract

Group B Streptococcus (GBS) is a major cause of neonatal morbidity and mortality. Serbia has not fully implemented preventive measures against GBS neonatal diseases. Therefore, we aimed to assess the maternal GBS colonisation and invasive neonatal disease rate, to reveal the trends of antimicrobial resistance and serotype distribution of GBS from various patient groups. Randomly selected non-invasive (n = 991) and all invasive GBS (n = 80) collected throughout Serbia from 2015 to 2020 were tested for antimicrobial susceptibility, capsular typing, and *hvgA* detection. Overall, 877/5621 (15.6%) pregnant women were colonised with GBS. Invasive GBS infections incidence in infants (0.18/1000 live births) showed a decreasing trend (0.3 to 0.1/1000 live births). Type III was overrepresented in infants with invasive infections (n = 35, 58.3%), whereas type V predominated among colonised adults (n = 224, 25.5%) and those with noninvasive (n = 37, 32.5%) and invasive infections (n = 8, 40%). The hypervirulent clone III/ST17 was highly associated with invasive infections (n = 28, 35%), particularly late-onset disease (n = 9, 47.4%), showing an increase from 12.3 to 14.8%. The GBS resistance to erythromycin and clindamycin was 26.7% and 22.1%, respectively, with an upward trend. The emergence of the hypervirulent clone III/ST17 and the escalation in GBS resistance highlight an urgent need for continuous monitoring of GBS infections.

## Introduction

*Streptococcus agalactiae* (Group B *Streptococcus*, GBS) is a leading cause of neonatal morbidity and mortality worldwide. It also represents an important opportunistic agent in pregnant women and non-pregnant adults, especially in the elderly or people with underlying medical conditions^1^. GBS may be a part of gastrointestinal and genital microbiota in 10–30% of healthy adults, including pregnant women^2^. In neonates and infants, GBS is mainly presented as two distinct syndromes: early-onset disease (EOD), associated with bacteremia within the first six days of life, and late-onset disease (LOD) accounting for most of the meningitis cases between the 7th and 89th day after birth^3^. The major risk factor for EOD is maternal rectovaginal colonisation and consequent vertical transmission during pregnancy. However, risk factors for LOD are not as well-characterised as for EOD. For LOD, infants may acquire GBS via vertical or horizontal transmission from colonised household contacts or the community^4,5^. In adults, GBS often presents as urogenital infections, bacteremia, skin and soft tissue, joint or bone infection, or pneumonia^6^.

The capsular polysaccharide (CPS) is the major virulence factor conferring anti-phagocytic properties of GBS. Based on the CPS composition, GBS has been classified into 10 distinct serotypes (Ia, Ib, and II–IX)^1^. Types Ia, II, III, and V accounted for 80–90% of all United States and European GBS clinical isolates^7^. Importantly, a highly virulent GBS clone III/ST17 has been reported as the main cause of the invasive neonatal disease responsible for up to 70% cases of meningitis^8^.

GBS is still uniformly sensitive to ß-lactam antibiotics, although since 2008 several reports of reduced susceptibility of GBS to penicillin have been reported^9,10^. For penicillin-allergic patients with low risk to anaphylaxis, cefazolin is the next alternative drug. Clindamycin given intravenously is recommended for penicillin-allergic patients at high risk of anaphylaxis. Intravenous vancomycin remains the only option for intrapartum antibiotic prophylaxis (IAP) in women with a high-risk penicillin allergy, and GBS isolate unsusceptible to clindamycin^11^. In recent years, increasing GBS resistance to erythromycin clindamycin, and fluoroquinolones, as well as high-level resistance (HLR) to gentamicin and decreased susceptibility to vancomycin have been reported worldwide^12–14^. Furthermore, GBS isolates with HLR to gentamicin have emerged in the United Kingdom and France^15,16^. Therefore, routine screening for HLR to gentamicin is important, although the broader clinical significance of this type of resistance is uncertain. Clinical recommendations and guidelines for the management of neonates with suspected or proven early-onset sepsis suggest penicillin-aminoglycoside combination therapy, although the synergistic effect of these antibiotics is still under debate^17,18^.

Pulsed-field gel electrophoresis (PFGE) is a useful genotyping method for bacterial strains with sufficient discriminatory power. It has been successfully used to characterise and distinguish specific clones among GBS isolates^19^.

In Serbia, limited data on the trends of the molecular epidemiology of circulating GBS isolates are available. Currently, there are no national guidelines for the prevention and treatment of invasive GBS diseases in infants. Screening-based strategy and risk-based approach are partially followed in Serbia, whereas, active Nationwide surveillance of invasive GBS infections in newborns has not been implemented. Therefore, the aims of the present study were: (1) to assess the GBS rectovaginal colonisation rate in pregnant women and the incidence of invasive GBS infections; (2) to determine CPS types distribution and changes over time in GBS circulating in Serbia; (3) to analyse genetic relatedness of invasive GBS isolates, and to reveal the proportion and the trend of the hypervirulent clone III/ST17; (4) to analyse the genetic relatedness of invasive GBS isolates, and (5) to evaluate antimicrobial susceptibility patterns and resistance determinants of invasive and non-invasive GBS strains.

## Results

### Bacterial isolates and colonisation

In the present study, a total of 80 invasive and 991 non-invasive GBS were analysed. Detailed specimen distribution within patient groups is shown in Table 1. During the study period**,** a total of 5621 pregnant women were screened for the GBS rectovaginal colonisation. The overall colonisation rate was found to be 15.6% (95% Cl 15.5–15.7), ranging from 12 to 17% in 5 years. Sixty out of 80 collected invasive GBS were isolated from infants (75.0%), constituting the overall incidence of 0.18 per 1000 live births (95% CI 0.1–0.2 per 1000). In addition, the incidence of EOD (n = 41) and LOD (n = 19) was 0.12 (95% CI 0.1–0.2 per 1000) and 0.05 (95% CI 0.0–0.1 per 1000), respectively. During the study period, there was a slight decrease in the prevalence of invasive GBS infections in infants, without significance (0.3 to 0.1 cases per 1000 live births; *p* = 0.351). Twenty out of 80 patients with invasive GBS infections were adults (25%). Blood culture was the most frequent specimen obtained from patients with invasive infections (68/80, 85%). Five out of eight CSF (62.5%) and both samples of synovial liquid (100%) were obtained from LOD cases. The sample types distribution within patient groups is presented in Table 1. The sepsis was the most common clinical manifestation among EOD, LOD, and adult invasive cases, diagnosed in 80.5%, 58%, and 90%, respectively.

**Table 1:** Specimen distribution of 1071 invasive and non-invasive isolates of Group B streptococci within patient groups.

Patient group	Invasive GBS isolates: N (%)			Non-invasive GBS isolates: N (%)		
	Children		Adults	Pregnant women—asymptomatic colonisation	Non-pregnant adults—urogenital infections	∑
Specimen type	EOD	LOD				
Blood	37 (90.2)	12 (63.2)	19 (95)			68 (6.3)
CSF	2 (4.9)	5 (26.3)	1 (5)			8 (0.8)
Pleural fluid	2 (4.9)					2 (0.2)
Synovial fluid		2 (10.5)				2 (0.2)
Rectovaginal swab				877 (100)		877 (81.9)
**Urogenital specimens**						
Genital swab					94 (82.5)	114 (10.6)
Urine					20 (17.5)	
∑	41 (100)	19 (100)	20 (100)	877 (100)	114 (100)	1071 (100)

*CSF* cerebrospinal fluid, *EOD* early-onset disease, *LOD*, late-onset disease, *GBS* Group B streptococcus.

Out of 114 GBS isolated from patients with non-invasive diseases, genital infections were more prevalent than urinary tract infections among adult patients with non-invasive diseases (82.5% vs 17.5%), as shown in Table 1.

### Capsular types distribution

**Figure 1:**
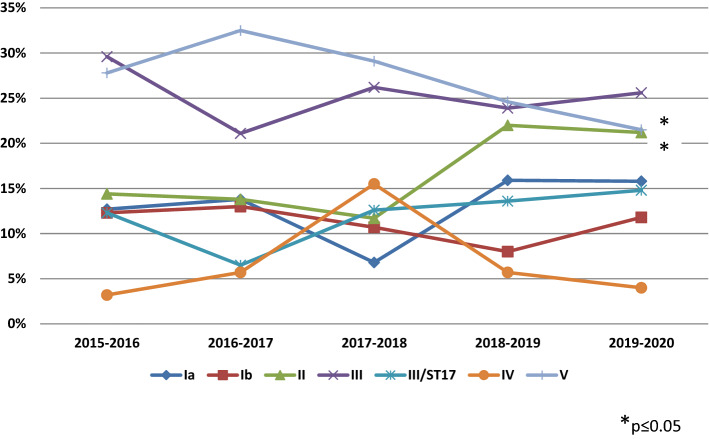
Trends of the capsular polysaccharide types distribution and the hypervirulent clone III/ST17 among 1071 invasive and non-invasive isolates of Group B streptococci in Serbia during a 5-year period.

In the present study, CPS types were assigned to all tested GBS isolates (n = 1071), and six types were detected (Ia, Ib, II to V). Overall, the most common types were V (26%) and III (25.8%), followed by types II (17.8), Ia (13.9%), Ib (11%), and IV (5.5%). The distribution of the CPS types among different patient groups is summarised in Table 2. Type III (35/60, 58.3%) was dominant among invasive GBS isolates recovered from infants, while the remaining types accounted together for 41.7%. CPS type III was more common among invasive isolates than among non-invasive ones (51.3% vs 23.7%; *p* < 0.01). Contrary to this, type II was more prevalent in non-invasive than invasive isolates (19% vs 3.8%, *p* < 0.01). Besides, type III was the most common in both EOD (19/41, 46.3%) and LOD cases (16/19, 84.2%), with a significantly higher proportion in LOD (*p* < 0.01). Additionally, among invasive cases, types Ia and II were exclusively represented in EOD cases. Otherwise, type V was dominant in adult patients with invasive infections (8/20, 40%), colonised pregnant women (224/877, 25.5%), and non-pregnant adult patients with urogenital infections (37/114, 32.5%). During the study period, several changes in the CPS types distribution were registered, as shown in Fig. 1. CPS type II increased significantly from 14.4 to 21.2% (p(CA) = 0.005), whereas type V decreased from 27.8 to 21.5% (p(CA) = 0.033). Besides, the significant decrease in the trend of the CPS type V from 27.4 to 21.2% was also seen among asymptomatically colonised pregnant women (p(CA) = 0.0327). Trends of the CPS types among colonising pregnant women and adults with non-invasive infections are presented in Supplementary Data—Figure S1, S2. The calculated potential of causing invasive disease showed that only CPS type III is likely to cause invasive disease (OR = 4.47, 95% Cl 2.617–7.649), while other types had OR values below 1 (range: 0.21–0.74). LOD cases showed the highest homogeneity of the CPS types distribution among all patients groups (SID = 0.199, 95% CI 0.135–0.413).

**Table 2:** Distribution of capsular polysaccharide types and hypervirulent clone III/ST17 among 1071 invasive and non-invasive isolates of Group B streptococci within different patient groups.

Patient group	Invasive GBS isolates: N (%)				Non-invasive GBS isolates: N (%)			
CPS type	EOD	LOD	Adults	∑	Pregnant women—asymptomatic colonisation		Non-pregnant adults—urogenital infections	∑
Ia	6 (14.6)	–	1 (5)	7 (8.8)	128 (14.6)		14 (12.3)	142 (14.3)
Ib	4 (9.8)	1 (5.3)	2 (10)	7 (8.8)	96 (10.9)		15 (13.2)	111 (11.2)
II	3 (7.3)	–	–	3 (3.8)	175 (20)		13 (11.4)	188 (19)
III/non-ST17	3 (7.3)	7 (36.8)	3 (15)	13 (16.3)	110 (12.5)		17 (14.9)	127 (12.8)
III/ST17	16 (39)	9 (47.4)	3 (15)	28 (35)	99 (11.3)		9 (7.9)	108 (10.9)
IV	1 (2.4)	1 (5.3)	3 (15)	5 (6.3)	45 (5.1)		9 (7.9)	54 (5.4)
V	8 (19.5)	1 (5.3)	8 (40)	17 (21.3)	224 (25.5)		37 (32.5)	261 (26.3)
∑	41 (100)	19 (100)	20 (100)	80 (100)	877 (100)		114 (100)	991 (100)

*CPS* capsular polysaccharide, *EOD* early-onset disease, *LOD* late-onset disease, *GBS* Group B streptococcus.

### Detection of hypervirulent clone

The *hvgA* gene detection was used for the identification of the hypervirulent ST17 clone. Overall, the *hvgA* gene was detected in 13.3% of isolates (n = 142), with the predominance in invasive over non-invasive isolates (37.5% vs 11.3%; *p* < 0.01). The majority of the ST17-positive GBS were detected among CPS type III (n = 136; 95.7%), while six remaining isolates belonged to the CPS type IV (4.3%). The hypervirulent clone type III/ST17 was more prevalent in invasive compared to non-invasive isolates (35% vs 10.9%; *p* < 0.01), as shown in Table 2. Additionally, the genotype III/ST17 was more prevalent in LOD compared to EOD cases (47.4% vs 39%, *p* = 0.58). Isolates of type IV harbouring the *hvgA* gene were detected in both invasive (n = 2) and non-invasive GBS (n = 4). Also, during the study period, a slight increase of the hypervirulent clone III/ST17 was detected from 12.3% in 2015 to 14.8% in 2020. However, significance was not observed (*p* = 0.206). Furthermore, a significant increase in the prevalence of the hypervirulent clone from 15 to 77.8% was seen among neonates with invasive infections (p(CA) = 0.0040) (Supplementary Data—Figure S3), as well as among overall GBS isolates of the CPS type III (41.7 to 57.9%; p(CA) = 0.0082). The overall proportion of the hypervirulent clone III/ST17 per year is shown in Supplementary Table S1.

### Pulsed-field gel electrophoresis

**Figure 2:**
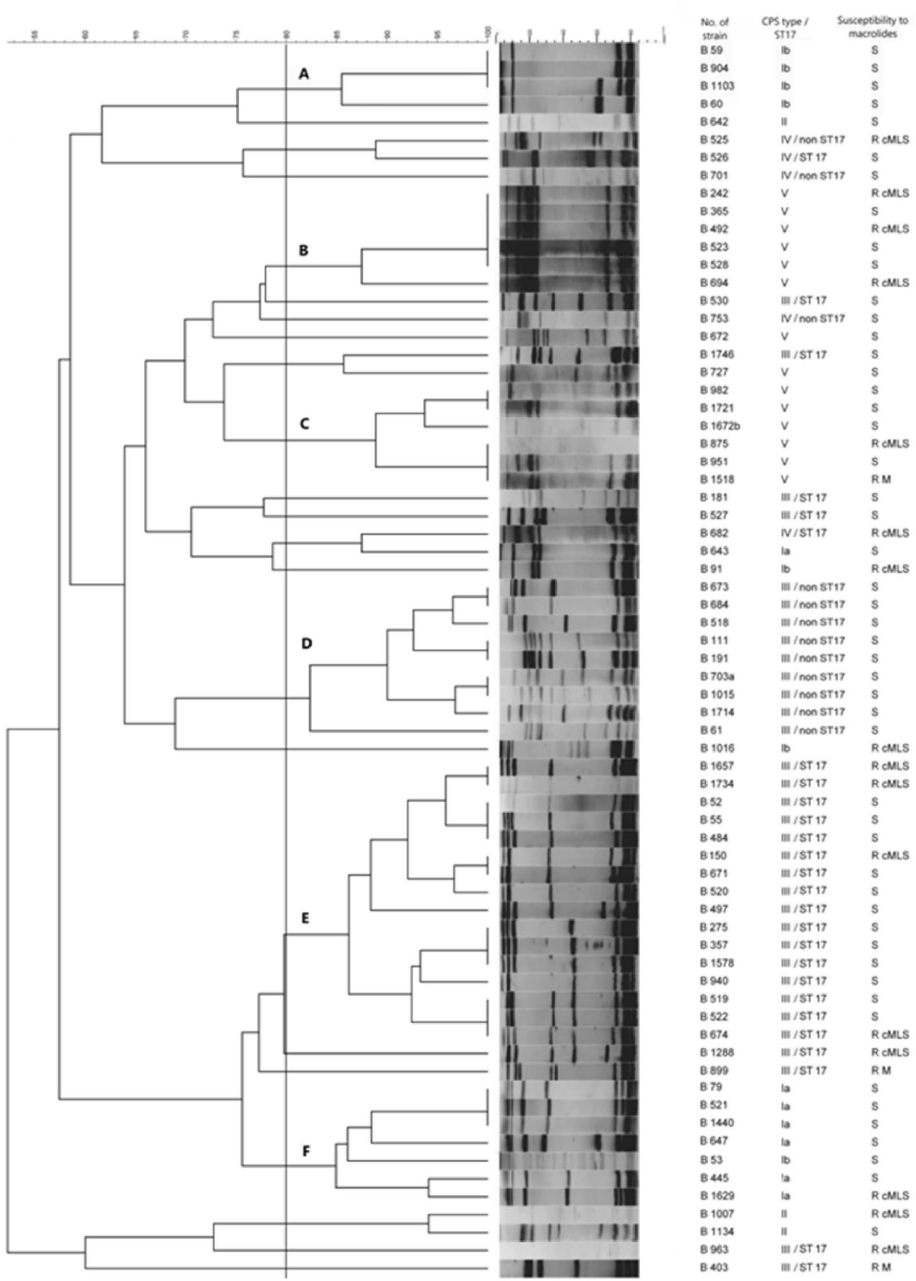
Dendrogram showing the genetic relationship of 69 invasive isolates of Group B streptococci established from PFGE patterns obtained after SmaI digestion. Dendrogram and PFGE patterns of SmaI-digested chromosomal DNA of Group B streptococci and association with capsular polysaccharide types, susceptibility to macrolides, macrolide resistance phenotypes, and the number of isolates belonging to these genotypes. The dendrogram was constructed with BioNumerics software, with 1.5% position tolerance, by using the UPGMA algorithm and Dice similarity coefficients. The clusters (clusters A, B, C, D, E, and F) contained isolates with similarity coefficients greater than 80% (indicated by the vertical line). Full-length gels are presented in Supplementary Data—Figures S4, S5, S6.

Overall, 47 different PFGE patterns were identified among the 69 tested invasive isolates (SID = 0.971, 95% CI 0.955–0.971), indicating that the analysed collection was genetically very diverse. Obtained profiles were grouped into six PFGE clusters (assigned as A to F) containing at least three isolates. Clustered GBS accounted for 69.6% (n = 48) of the tested isolates, while the remaining 21 isolates were represented as minor PFGE groups (n = 2 isolates) or as unique profiles. The dendrogram depicting the relationship between PFGE patterns is shown in Fig. 2. Each of the five PFGE clusters (A, B, C, D, and E) comprised exclusively of isolates of the same CPS type, while cluster F, composed of the CPS type Ia (n = 6) and one isolate with CPS type Ib. The composition of these PFGE clusters indicates a very good predictive power of the PFGE-based genotypes for the CPS types (WC = 0.965, 95%Cl 0.918–1.000). Conversely, as presented in Fig. 2, PFGE cluster F and minor groups consisted of GBS isolates with different CPS types pointing that several genetic lineages share the same CPS type. The most homogeneous CPS type was V (SID = 0.785, 95% CI 0.561–0.836), although these isolates were grouped into two clusters (B and C). The largest PFGE cluster E (n = 16), was composed of hypervirulent clone III/ST17 (SID = 0.875, 95% Cl 0.687–0.875), whereas the remaining isolates of the same genotype clustered in the minor PFGE group (n = 1) or as unique profiles (n = 7). One isolate with CPS type IV and ST17 was grouped with the isolate IV/non-ST17 in a minor group while the other strain of the same genotype clustered with GBS III/ST17. Interestingly, all GBS isolates with the genotype III/non-ST17 were clustered together (cluster D), separately from GBS III/ST17.

### Antimicrobial susceptibility testing

Antimicrobial susceptibility testing of 1071 GBS was done by disk diffusion and gradient test. All GBS isolates were susceptible to penicillin, vancomycin, norfloxacin, and chloramphenicol. Penicillin MICs ranged from 0.006 to 0.094 μg/ml, with a mode value of 0.016 μg/ml, MIC_50_ of 0.023 μg/ml, and MIC_90_ of 0.064 μg/ml. The HLR to gentamicin was not detected among the tested isolates. Overall, the resistance rate of the tested GBS to erythromycin, clindamycin, and tetracycline was 26.7%, 22.1%, and 85.2%, respectively. The antimicrobial resistance rates within different patient groups are presented in Table 3. The most common macrolide-resistant phenotype was cMLS (n = 174; 60.8%), followed by iMLS (n = 63; 22%) and M phenotype (n = 49; 17.1%). The genetic background of cMLS and iMLS phenotypes was associated with the presence of the *ermB* and *ermA* genes, respectively, while the *mefA* gene mediated the M phenotype. Five strains with cMLS phenotype harboured both *ermB* and *mefA* genes. The MICs of erythromycin and clindamycin were ≥ 256 μg/ml in *ermB*-positive strains. Most of the macrolide-resistant strains were associated with CPS types V (36.4%) and III (23.4%), while the remaining types accounted for 40.2%. All macrolide-resistant isolates were found to be co-resistant to tetracycline. Resistance to tetracycline was encoded mainly by the *tetM* gene (n = 726; 79.5%), followed by *tetO* (n = 186; 20.4%), and *tetL* (n = 1; 0.1%). The association of the *tetM* gene and other tetracycline resistance genes*, tetO* and *tetL* was found in 5.2% (n = 48) and 0.1% (n = 1), respectively. In two tetracycline susceptible isolates, the *tetM* gene was detected. The distribution of macrolide and tetracycline resistance genes within different CPS types is presented in Table 4. During the study period, erythromycin and clindamycin resistance rates increased from 19.7 to 29.2% (p(CA) = 0.0033) and from 17.3 to 24.1% (p(CA) = 0.0278), respectively. Also, there was an increase in the overall rate of GBS resistance to tetracycline, from 82 to 90.9% (p(CA) = 0.0111). Data concerning antimicrobial resistance per year are shown in Supplementary Table S2. Macrolide-resistant GBS isolates subjected to the PFGE analysis yielded diverse PFGE profiles and thus were assigned to four different clusters (B, C, E, and F), minor PFGE groups, and unique profiles, indicating their genetic diversity. A substantial proportion of clusters B and C, composed of CPS type V, were macrolide-resistant, 50% and 33.3%, respectively. Contrary to the hypervirulent GBS III/ST17, all isolates of the genotype III/non-ST17 were susceptible to macrolides (cluster D).

**Table 3:** Distribution of erythromycin, clindamycin and tetracycline resistance rates and macrolide resistance phenotypes in 1071 invasive and non-invasive isolates of Group B streptococci within different patient groups.

Patients group		Invasive GBS isolates (n = 80) N (%)				Noninvasive GBS isolates (n = 991) N (%)		
Antibiotic resistance		EOD	LOD	Adults	∑	Pregnant women—asymptomatic colonisation	Non-pregnant adults—urogenital infections	∑
Erythromycin		12 (29.3)	6 (31.6)	5 (25)	23 (28.8)	233 (26.6)	30 (26.3)	263 (26.5)
**Phenotype**								
cMLS		7 (58.3)	4 (66.7)	3 (60)	14 (60.9)	145 (62.2)	15 (50)	160 (60.8)
iMLS		3 (25)	1 (16.7)	–	4 (14.4)	47 (20.2)	12 (40)	59 (22.4)
M		2 (16.7)	1 (16.7)	2 (40)	5 (21.7)	41 (17.6)	3 (10)	44 (16.7)
Clindamycin		10 (24.4)	5 (26.3)	3 (15)	18 (22.5)	192 (21.9)	27 (23.7)	219 (22.1)
Tetracycline		36 (87.8)	13 (68.4)	16 (80)	65 (81.3)	754 (86)	94 (82.5)	848 (85.6)
∑		41 (100)	19 (100)	20 (100)	80 (100)	877 (100)	114 (100)	991 (100)

*EOD* early-onset disease, *LOD* late-onset disease, *GBS* Group B streptococcus.

**Table 4:** Proportion of macrolide and tetracycline resistance genes detected in 1071 isolates of Group B streptococci within capsular types.

CPS type	No. of isolates	No. of MR isolates	Macrolide resistance genes N (%)			No. of TR isolates	Tetracycline resistance genes N (%)		
			*ermA*	*ermB*	*mefA*		*tetM*	*tetO*	*tetL*
Ia	149	29 (19.5)	5 (3.4)	10(6.7)	20 (13.4)	134 (90)	134 (90)	–	–
Ib	118	18 (15.3)	2 (1.6)	16 (13.5)	2 (1.6)	92 (78)	69 (58.5)	23 (19.5)	–
II	191	48 (25.1)	5 (2.6)	39 (20.4)	6 (3.2)	170 (89)	111 (58.1)	59 (30.9)	–
III	276	67 (24.3)	10 (3.6)	45 (16.3)	22 (7.8)	224 (81.2)	153 (55.4)	70 (25.4)	1 (0.4)
IV	59	20 (33.9)	4 (6.8)	17 (28.8)	–	55 (93.2)	40 (67.8)	15 (25.4)	–
V	278	104 (37.4)	20 (7.2)	81 (29.1)	6 (2.2)	238 (85.6)	210 (75.5)	27 (9.7)	–

*CPS* capsular polysaccharide, *MR* macrolide resistance, *TR* tetracycline resistance.

## Discussion

The current study provides epidemiological and molecular insights into the GBS isolated from asymptomatic colonised pregnant women, as well as infants and non-pregnant adults with infections, during 5 years. This is the first study describing trends in antimicrobial resistance and genotypes distribution of the invasive and non-invasive GBS in Serbia.

The estimated prevalence of maternal GBS rectovaginal colonisation in the current study (15.6%) corresponded to the previously reported colonisation rate in Serbia in 2015/2016 (15%)^20^. According to an already published report, the worldwide GBS colonisation rate among pregnant women varies geographically, ranging from 11.1% in Asia to over 30% in Africa^21^. Global maternal GBS colonisation rate was estimated to be around 18%, while prevalences in European countries were slightly lower (15.2–15.4%)^21^.

In the present study, the overall incidence of invasive GBS infections in infants (0.18 per 1000 live births) was estimated to be more than 2.5 fold lower than the global burden of invasive GBS disease in newborns (0.49–0.53 per 1000 live births)^22^. However, the evaluated incidence is similar to that inferred for Crete (Greece) over a 22-year period where overall GBS incidence was 0.17 per 1000 live births^23^. In the present study, a slight decline in the incidence of both EOD and LOD cases was registered during the observed 5-year period. Preventive measures and recommendations had a significant impact on the rates of invasive GBS, though LODs are not affected by prenatal and intrapartum prevention strategies^24^. Therefore, low incidence and decreasing trends of invasive GBS infections in newborns might be partially explained by the lack of a national surveillance program of invasive GBS infections in Serbia.

Although several changes in the distribution of CPS types were detected during the study period, type III remained dominant in the paediatric population, while type V was the most common among pregnant women and non-pregnant adults. Notably, the hypervirulent clone III/ST17 accounted for more than one-third of all invasive isolates, almost half of the LOD, and two-third of the meningitis cases. Such findings were confirmed by calculating high invasive OR value for CPS type III, indicating its potential to cause invasive disease. Similar findings with OR values between 1.8–4.2 for CPS type III were reported elsewhere^25^. Furthermore, a worrisome incremental trend of the hypervirulent clone CPS III/ST17 was noticed, as well as an emergence of the CPS IV/ST17. The overrepresentation of the hypervirulent type III/ST17 in LOD and meningitis cases has been well-documented worldwide^26,27^. Thus, Manning et al. found that ST17 strains were more likely to cause meningitis than all other types^28^. This can be explained by the presence of surface protein HvgA, a factor that is normally expressed by ST17 strains and is associated with an increased ability to cross the blood–brain barrier^29^. Neonatal serotypes coverage of the trialled conjugated pentavalent (Ia, Ib, II, III, and V) and trivalent (Ia, Ib, III) vaccines were assessed to be 96.6% and 76.6%, respectively. Assumed coverage is similar to those reported by Lu et al.^30^.

Pregnant women colonised with GBS are considered to be the main reservoirs of invasive strains in neonates. Therefore, several studies found that colonising GBS isolates had serotype distributions that closely mirrored those of invasive neonatal GBS diseases in the same geographic area^31,32^. However, in the present study, the hypervirulent clone III/ST17 only accounted for 11% of maternal GBS colonisation. We found that the genotypic characteristics of colonised GBS strains in pregnant women and invasive neonatal strains were quite different. Such switching of molecular characteristics has been found during vertical transmission^33,34^.

In the current study, CPS type V was shown to be the most homogenous type. The obtained finding is probably the result of its clonal propagation which is in accordance with previously published reports^35–37^. Interestingly, based on the PFGE banding patterns, genotype III/ST17 was less homogenous. The obtained finding led to the hypothesis that PFGE may distinguish different ST17 lineages^37,38^. Expectedly, the PFGE analysis showed that multiple PFGE patterns might share the same CPS type, while one PFGE profile corresponds to a specific CPS type. In the present study, all PFGE clusters, except one were composed of the same CPS type. Similar findings showing that PFGE-based profiles show very good predictive power for the CPS type, while strains of the same type can be subdivided into several PFGE profiles were previously reported^38^. Two invasive GBS IV/ST17 profiled by PFGE showed that one isolate was grouped with type IV/ST17-negative and the other with III/ST17 strains, indicating the possibility of both previously reported genetic recombination events—the acquisition of the HvgA adhesin and the capsular switching, respectively^29^.

Although GBS strains with reduced susceptibility to penicillin have been reported, in the present study, all tested strains were susceptible to penicillin^9^. However, high rates of erythromycin, clindamycin, and tetracycline resistance were detected, with rising trends during the observed period. Resistance to erythromycin and clindamycin significantly increased from 2015 to 2020, with an increment of 9.5% and 6.8%, respectively. Moreover, the overall macrolide and clindamycin resistance rates were higher compared to the previous report on GBS in Serbia, 26.7% versus 23.1%, and 22.1% versus 21.3%, respectively^20^. Accordingly, the Center for disease prevention and control marked clindamycin resistant GBS as one of the Antibiotic Resistance Threats in the United States^39^. In the same report, these strains, together with the erythromycin-resistant GBS, are underlined as major causes of infections, limiting prevention and treatment options for various patient groups with severe penicillin allergies.

The majority of the macrolide-resistant GBS expressed cMLS phenotype, encoded by the *ermB* gene. The CPS type V dominated among GBS with cMLS phenotype, reflecting clonal spread with the selective advantage of antimicrobial resistance. The obtained results are consistent with those of the previously published studies conducted in Serbia, South Corea, and England^20,40,41^. Although PFGE profiling of invasive GBS isolates showed that several lineages contributed to the macrolide resistance, the clonal spread of CPS types V and III harbouring gene encoding cross-resistance to macrolide, lincosamide, and streptogramin were found to be an important driving force for macrolide resistance. Besides, the isolates of the hypervirulent clone III/ST17 were more resistant to macrolides compared to GBS isolates III/non-ST17, which increasing trend might impact the overall increase in macrolide resistance. In the present study, PFGE clustering was an excellent predictor of CPS types, but not of macrolide-resistant GBS. A high percentage of tetracycline resistance was also detected with a steady rise during the study period. The resistance mechanism was mostly mediated by ribosomal protection proteins, while efflux pumps were uncommon. These findings are in line with a previously published report^40,42^. Most of the macrolide-resistant isolates were co-resistant to tetracycline and were associated with CPS type V and III. Obtained results are in accordance with a previous report that found a correlation between serotypes III and V and macrolide and tetracycline resistance^43^.

Limitations of the study are the possibility of underreporting of invasive GBS infections due to the lack of a national surveillance system and false-negative cases which might arise from the antibiotic treatment before the specimen sampling. Also, the strategies for preventing GBS infections in newborns are partially implemented in Serbia due to the absence of a national guideline for the prevention and treatment of neonatal diseases.

## Conclusion

The current study aimed to provide overall data on the prevalence of maternal colonisation and newborn infections as well as genotypic changes of GBS isolates in 5 years in Serbia. Our data show that the prevalence of GBS rectovaginal colonisation among pregnant women is similar to other European reports. The prevalence of infants with invasive infections is very low and probably underestimated. Overall, the most common CPS types circulating in Serbia are type III and V. Their genetic homogeneity indicated successful clonal propagation. CPS type III has shown the highest potential to cause invasive disease. Particularly, the hypervirulent type III/ST17 showed association with invasive neonatal isolates, especially LOD. Penicillin remains the antibiotic of choice for IAP and treatment of GBS infections, with recommended susceptibility testing for macrolides and lincosamides before administration to patients hypersensitive to penicillin. Consequently, the results of the present study, indicate an urgent need for the introduction of continuous national surveillance of invasive GBS infections, as well as recommendations for maternal colonisation screening and management.

## Methods

### Bacterial strains

A total of 1071 nonredundant invasive and non-invasive GBS isolates recovered throughout Serbia from January 2015 till January 2020 were included in the study. Isolates were obtained from 10 microbiological laboratories located in seven regional hospitals throughout Serbia, three gynaecology and obstetrics clinics and two major paediatric clinics in the country. Bacterial isolation and identification were carried out during the routine work in hospital microbiological laboratories. GBS isolates, along with the corresponding clinical data, were sent to the National reference laboratory (NRL) for streptococci for further analysis. Adults were defined as patients aged 18 years and older, while children were defined as patients younger than 18 years. During the study period, participating laboratories were collected all invasive isolates and randomly selected non-invasive GBS. A total of 80 invasive strains were isolated from the sterile sites (e.g. blood, cerebrospinal fluid, and synovial fluid), while 991 non-invasive isolates were recovered from pregnant women with asymptomatic GBS colonisation (n = 877) and non-pregnant women with urogenital infections (n = 114). The estimation of the incidence of invasive neonatal infections was done based on the total number of the reported cases for a specified period and the official data of the number of live births in the respective area of the Republic of Serbia from 2015 to 2020, obtained from the Statistical Office of the Republic of Serbia^44^.

During the study period, a total of 5621 randomly selected pregnant women were screened for the GBS rectovaginal colonisation. Briefly, rectovaginal swabs were taken from pregnant women during 36–37 week of the pregnancy (flocked-swab, Copan, Italy) and placed in selective Todd-Hewitt broth supplemented with colistin (10 mg/l) and nalidixic acid (15 mg/l) (Lim broth, Becton Dickinson, USA) and incubated overnight at 35 °C. Strains were subcultured on chromID Strepto B (BioMerieux, France) under aerobic conditions at 35 °C for 24 h. Positive isolates were confirmed as GBS with commercial GBS latex-agglutination assays (Slidex Strepto B, bioMérieux, France) and stored in the Skim milk stock medium at − 70 °C.

All isolates were identified based on colony morphology, β-haemolysis, Gram staining, catalase test, and commercial GBS latex-agglutination assays (Slidex Strepto B, bioMérieux, France). PCR amplification of a species-specific regulatory gene *dltR*, was used as molecular confirmation of GBS^45^.

### Molecular typing analysis

Capsular typing was performed by multiplex PCR, as described previously^46,47^. The hypervirulent ST17 clone was identified by the detection of hypervirulent GBS adhesin (hvgA) encoding gene by PCR^45^. Molecular profiling of invasive GBS was done by PFGE in agarose plugs, as previously described^38,48^. Total bacterial DNAs of 69 invasive GBS, randomly selected with respect to the CPS type, were isolated and digested with SmaI (Thermo Scientific) enzyme for 18–24 h at 25 °C^49^. The fragments were separated by PFGE in 1.2% agarose gels in a CHEF-DR II system (Bio-Rad Laboratories, Hercules, CA) with pulse times of 2 to 30 s for 23 h at 4 °C and 6 V/cm. The restriction profiles were analysed using the BioNumerics software v7.6 (Applied Maths, Belgium). The Dice similarity coefficient was used to compare the banding profiles, and a dendrogram was constructed using the unweighted-pair group method with arithmetic averages (UPGMA) with a tolerance coefficient of 1.5%. Isolates with similarities more significant than 80% were considered closely related and clustered together.

### Antimicrobial susceptibility testing

Antimicrobial susceptibility testing (AST) to penicillin (10U), norfloxacin (10 µg), vancomycin (5 µg), erythromycin (15 µg), clindamycin (2 µg), chloramphenicol (30 µg), and tetracycline (30 µg) was performed by the disk diffusion test (Bio-Rad Laboratories Ltd., UK), according to the European Committee on Antimicrobial Susceptibility Testing (EUCAST)^50^. Minimal inhibitory concentrations (MICs) of penicillin were determined for all GBS isolates using Etest (bioMérieux, France), whereas MICs of erythromycin and clindamycin were evaluated for macrolide-resistant strains. Susceptibility categories were interpreted according to the EUCAST guidelines^50^. *Streptococcus pneumoniae* ATCC 49619 was used as a control. The High-Level Aminoglycoside Resistance (HLAR) was screened according to the recommendations of the French Society for Microbiology^51^. Macrolide resistance phenotypes were determined based on the double-disk diffusion test using erythromycin (15 μg) and clindamycin (2 μg) disks, as previously described^50^. Erythromycin resistant isolates were tested for the presence of the macrolide-resistant genes (*ermA, ermB,* and *mefA)*, while all GBS were screened for the tetracycline resistance determinants (*tetO, tetM, tetK,* and *tetL)* using previously published protocols^52^.

### Statistical analysis

The SPSS (version 20.0 for Windows) is a software package used for statistical analysis. The χ2 and Fisher tests were used to assess possible associations and differences between the variables. Besides, the Cochran-Armitage (CA) linear trends test was used. A *p* ≤ 0.05 value was considered to be significant. Simpson’s index of diversity (SID) with 95% confidence intervals (95% CI) was used to evaluate the diversity of the tested isolates^53^. The concordance between typing methods was calculated using the Wallace coefficient (WC)^54^. All calculations were done using the freely available online tool Comparing Partitions located at www.comparingpartitions.info. The potential of causing invasive disease for each CPS type was calculated based on the invasive odds ratio (OR)^25^. OR > 1 indicates an increased probability that a CPS type causes invasive disease, OR < 1 indicates a reduced probability, while OR = 1 indicates the possibility of causing invasive disease and recovering is equal. Information regarding the Serbian population and the number of live births per year in the study period (2015–2020), was obtained from the Statistical Office of the Republic of Serbia^44^.

### Ethics approval

This study was approved by the Ethical Committee of the Medical Faculty University of Belgrade, Serbia (No.1550/II-10). All methods were performed following the relevant guidelines and regulations. Patient identifiable information was anonymised in regional clinical laboratories and re-coded at the NRL for streptococci.

## Supplementary information

Supplementary Information.

## References

[1] Dutra VG (2014). *Streptococcus agalactiae* in Brazil: Serotype distribution, virulence determinants and antimicrobial susceptibility. BMC Infect. Dis..

[2] Brimil N (2006). Epidemiology of *Streptococcus agalactiae* colonisation in Germany. Int. J. Med. Microbiol..

[3] Shabayek S, Spellerberg B (2018). Group B streptococcal colonisation, molecular characteristics, and epidemiology. Front. Microbiol..

[4] Schrag SJ, Verani JR (2013). Intrapartum antibiotic prophylaxis for the prevention of perinatal group B streptococcal disease: Experience in the United States and implications for a potential group B streptococcal vaccine. Vaccine.

[5] Schuchat A (1998). Epidemiology of group B streptococcal disease in the United States: Shifting paradigms. Clin. Microbiol. Rev..

[6] Sendi P, Johansson L, Norrby-Teglund A (2008). Invasive group B streptococcal disease in non-pregnant adults: A review with emphasis on skin and soft-tissue infections. Infection.

[7] Martins ER (2017). *Streptococcus agalactiae* causing neonatal infections in Portugal (2005–2015): Diversification and emergence of a CC17/PI-2b multidrug resistant sublineage. Front. Microbiol..

[8] Poyart C (2008). Invasive group B streptococcal infections in infants, France. Emerg. Infect. Dis..

[9] Kimura K (2008). First molecular characterisation of group B streptococci with reduced penicillin susceptibility. Antimicrob. Agents Chemother..

[10] Jamrozy D (2020). Increasing incidence of group B streptococcus neonatal infections in the Netherlands is associated with clonal expansion of CC17 and CC23. Sci. Rep..

[11] Di Renzo GC (2015). Intrapartum GBS screening and antibiotic prophylaxis: A European consensus conference. J. Matern. Neonatal Med..

[12] Back EE, O’Grady EJ, Back JD (2012). High rates of perinatal group B Streptococcus clindamycin and erythromycin resistance in an Upstate New York Hospital. Antimicrob. Agents Chemother..

[13] Gygax SE (2006). Erythromycin and clindamycin resistance in group B streptococcal clinical isolates. Antimicrob. Agents Chemother..

[14] Raabe VN, Shane AL (2019). Group B Streptococcus (*Streptococcus agalactiae*). Microbiol. Spectr..

[15] Liddy H, Holliman R (2002). Group B Streptococcus highly resistant to gentamicin. J. Antimicrob. Chemother..

[16] Hays C (2016). Changing epidemiology of group B Streptococcus susceptibility to fluoroquinolones and aminoglycosides in France. Antimicrob. Agents Chemother..

[17] Puopolo KM, Benitz WE, Zaoutis TE (2018). Committee on Fetus and Newborn; Committee on Infectious diseases. Management of neonates born at ≥35 0/7 weeks’ gestation with suspected or proven early-onset bacterial sepsis. Pediatrics.

[18] Ruppen C, Lupo A, Decosterd L, Sendi P (2016). Is Penicillin plus gentamicin synergistic against clinical group B Streptococcus isolates? An in vitro study. Front. Microbiol..

[19] Skjaervold NK, Bergh K, Bevanger LS (2004). Distribution of PFGE types of invasive Norwegian group B streptococci in relation to serotypes. Indian J. Med. Res. Suppl..

[20] Gajic I (2019). Molecular epidemiology of invasive and non-invasive group B Streptococcus circulating in Serbia. Int. J. Med. Microbiol..

[21] Russell NJ (2017). Maternal colonisation with Group B Streptococcus and serotype distribution worldwide: Systematic review and meta-analyses. Clin. Infect. Dis..

[22] Madrid L (2017). Infant Group B streptococcal disease incidence and serotypes worldwide: Systematic review and meta-analyses. Clin. Infect. Dis..

[23] Vergadi E (2018). Changes in the incidence and epidemiology of neonatal group B Streptococcal disease over the last two decades in Crete, Greece. Infect. Dis. Rep..

[24] Johri AK (2006). Group B Streptococcus: Global incidence and vaccine development. Nat. Rev. Microbiol..

[25] Song JY, Lim JH, Lim S, Yong Z, Seo HS (2018). Progress toward a group B streptococcal vaccine. Hum. Vaccines Immunother..

[26] Joubrel C (2015). Group B streptococcus neonatal invasive infections, France 2007–2012. Clin. Microbiol. Infect..

[27] Creti R (2017). Neonatal Group B Streptococcus infections: Prevention strategies, clinical and microbiologic characteristics in 7 years of surveillance. Pediatr. Infect. Dis. J..

[28] Manning SD (2009). Multilocus sequence types associated with neonatal group B streptococcal sepsis and meningitis in Canada. J. Clin. Microbiol..

[29] Bellais S (2012). Capsular switching in group B Streptococcus CC17 hypervirulent clone: A future challenge for polysaccharide vaccine development. J. Infect. Dis..

[30] Lu B (2016). Molecular characteristics and antimicrobial resistance in invasive and non-invasive Group B Streptococcus between 2008 and 2015 in China. Diagn. Microbiol. Infect. Dis..

[31] Medugu N (2017). Group B streptococcal colonisation and transmission dynamics in pregnant women and their newborns in Nigeria: Implications for prevention strategies. Clin. Microbiol. Infect..

[32] Teatero S (2016). Clonal Complex 17 Group B Streptococcus strains causing invasive disease in neonates and adults originate from the same genetic pool. Sci. Rep..

[33] Björnsdóttir ES (2016). Changing epidemiology of group B streptococcal infections among adults in Iceland: 1975–2014. Clin. Microbiol. Infect..

[34] Li S (2018). Molecular characteristics of *Streptococcus agalactiae* in a mother–-baby prospective cohort study: Implication for vaccine development and insights into vertical transmission. Vaccine.

[35] Pillai P (2009). *Streptococcus agalactiae* pulsed-field gel electrophoresis patterns cross capsular types. Epidemiol. Infect..

[36] Brzychczy-Włoch M (2010). Genetic characterisation and diversity of *Streptococcus agalactiae* isolates with macrolide resistance. J. Med. Microbiol..

[37] Kardos S (2019). High prevalence of group B streptococcus ST17 hypervirulent clone among non-pregnant patients from a Hungarian venereology clinic. BMC Infect. Dis..

[38] Martins ER (2007). Analysis of Group B streptococcal isolates from infants and pregnant women in Portugal revealing two lineages with enhanced invasiveness. J. Clin. Microbiol..

[39] CDC (2019). Antibiotic Resistance Threats in the United States, 2019.

[40] Yoon IA (2015). Clinical significance of serotype V among infants with invasive group B streptococcal infections in South Korea. Int. J. Infect. Dis..

[41] Lamagni TL (2013). Emerging trends in the epidemiology of invasive group B streptococcal disease in England and Wales, 1991–2010. Clin. Infect. Dis..

[42] Lopes E (2018). Increasing macrolide resistance among *Streptococcus agalactiae* causing invasive disease in non-pregnant adults was driven by a single capsular-transformed lineage, Portugal, 2009 to 2015. Eurosurveillance.

[43] Tsai MH (2019). Molecular characteristics and antimicrobial resistance of Group B streptococcus strains causing invasive disease in neonates and adults. Front. Microbiol..

[44] Statistical Office of the Republic of Serbia. *Statistical Pocketbook of the Republic of Serbia* (accessed 06 May 2020); https://www.stat.gov.rs/en-US/oblasti/stanovnistvo/rodjeni-i-umrli/ (2020).

[45] Lamy MC (2006). Rapid detection of the “highly virulent” group B streptococcus ST-17 clone. Microbes Infect..

[46] Poyart C (2007). Multiplex PCR assay for rapid and accurate capsular typing of group B streptococci. J. Clin. Microbiol..

[47] Yao K (2013). Capsular gene typing of *Streptococcus agalactiae* compared to serotyping by latex agglutination. J. Clin. Microbiol..

[48] Kojic M, Strahinic I, Fira D, Jovcic B, Topisirovic L (2006). Plasmid content and bacteriocin production by five strains of *Lactococcus lactis* isolated from semi-hard homemade cheese. Can. J. Microbiol..

[49] Silva-Costa C, Ramirez M, Melo-Cristino J (2006). Identification of macrolide-resistant clones of *Streptococcus pyogenes* in Portugal. Clin. Microbiol. Infect..

[50] The European Committee on Antimicrobial Susceptibility Testing. *Breakpoint Tables for Interpretation of MICs and Zone Diameters, Version 10.0* (accessed 10 January 2020); http://www.eucast.org/clinical_breakpoints/ (2020).

[51] Société Française de Microbiologie. *Recommandations 2020, V.1.1 Avril* (accessed 01 may 2020); http://www.sfm-microbiologie.org/wp-content/uploads/2020/04/CASFM2020_Avril2020_V1.1.pdf/ (2020).

[52] Compain F (2014). Molecular characterisation of *Streptococcus agalactiae* isolates harboring small erm(T)-carrying plasmids. Antimicrob. Agents Chemother..

[53] Grundmann H, Hori S, Tanner G (2001). Determining confidence intervals when measuring genetic diversity and the discriminatory abilities of typing methods for microorganisms. J. Clin. Microbiol..

[54] Pinto FR, Melo-Cristino J, Ramirez M (2008). A confidence interval for the Wallace coefficient of concordance and its application to microbial typing methods. PLoS ONE.

