# Supplementary material for: Trends in molecular characteristics and antimicrobial resistance of group B streptococci: a multicenter study in Serbia, 2015–2020
Source: Sci Rep. 2021 Jan 12;11:540. doi: 10.1038/s41598-020-79354-3 (PMC7804007; doi:10.1038/s41598-020-79354-3)
Supplement: Supplementary file 1 — Supplementary Information. [file 41598_2020_79354_MOESM1_ESM.pdf]

Supplementary Information file

TITLE PAGE

Trends in molecular characteristics and antimicrobial resistance of group B streptococci: a multicenter study in Serbia, 2015–2020

Dusan Kekic[1*+], Ina Gajic[1+], Natasa Opavski[1], Milan Kojic[2], Goran Vukotic[2,3], Aleksandra Smitran[4], Lidija Boskovic[5], Marina Stojkovic[6], Lazar Ranin[1]

[1] Institute for Microbiology and Immunology, Medical Faculty, University of Belgrade, 11000 Belgrade, Serbia

[2] Institute of Molecular Genetics and Genetic Engineering, University of Belgrade, Belgrade, 11000 Belgrade, Serbia

[3] Faculty of Biology, University of Belgrade, Belgrade, 11000 Belgrade, Serbia

[4] Institute for Microbiology and Immunology, Medical Faculty, University of Banja Luka, 78000, Banja Luka, Bosnia and Herzegovina

[5] Department for Microbiology, University Hospital Center “Dr Dragisa Misovic”, 11000 Belgrade, Serbia

[6] Department for Microbiology, The Obstetrics and Gynaecology Clinic “Narodni Front”, 11000 Belgrade, Serbia

***Corresponding author:** Institute for microbiology and immunology, Medical Faculty, University of Belgrade, Dr Subotica no.1, 11000 Belgrade, Serbia; e-mail: dusan_vk@yahoo.com

[+] equally contributed

Supplementary Figure S1 - Trends of capsular types distribution of Group B streptococci among asymptomatically colonised pregnant women (n=877)

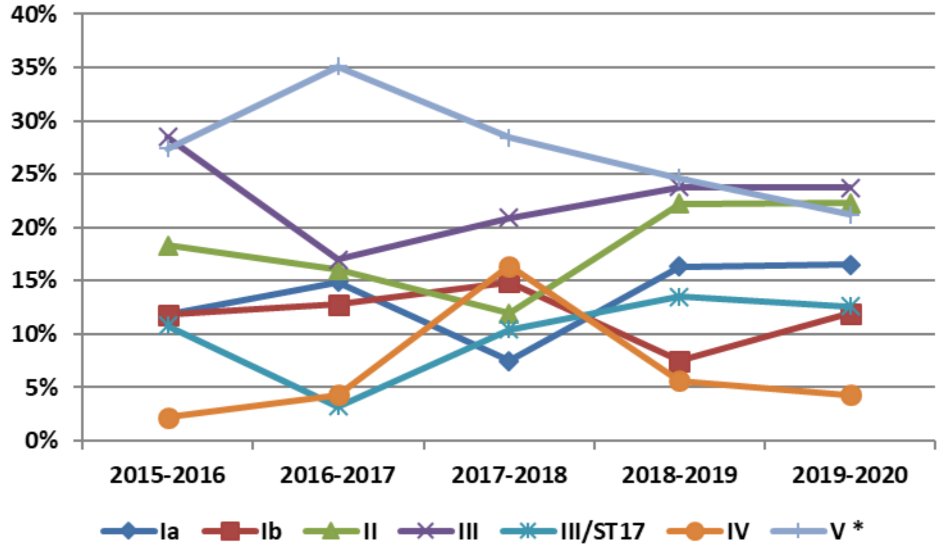

*Significant decrease in the trend of CPS type V (27.4% to 21.2%) during the study period (p (CA)=0.0327)

Supplementary Figure S2 - Trends of capsular types distribution of Group B streptococci among non-pregnant adults with urogenital infection (n=114)

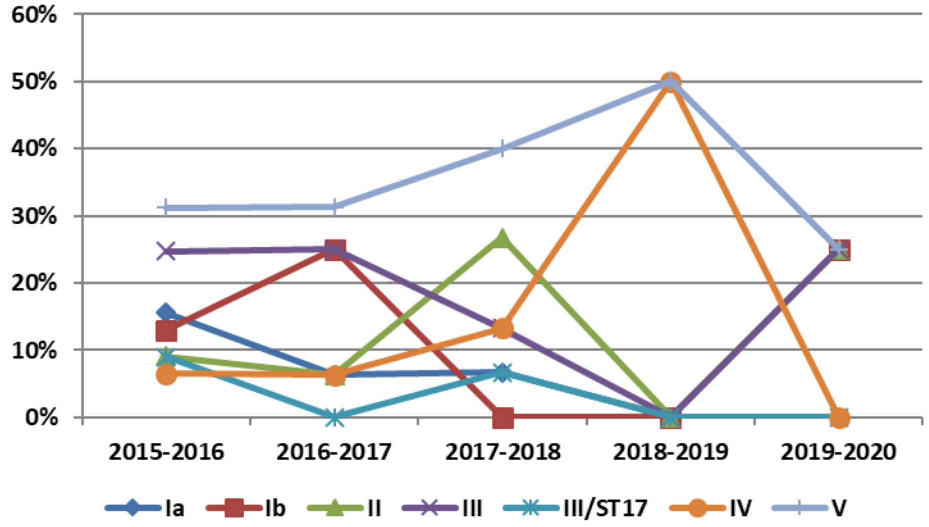

No significant changes in the trend of the CPS types distribution during the study period.

Supplementary Figure S3- Trends of capsular types distribution of Group B streptococci among neonates with invasive infections (n=60)

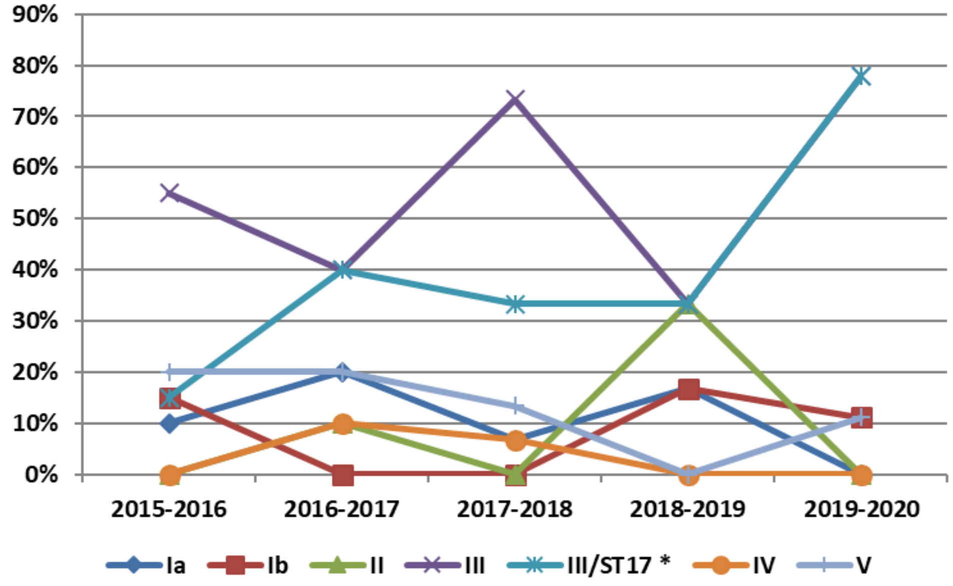

* Significant increase in the trend of the hypervirulent clone III/ST17 (15% to 77.8%) during the study period (p(CA)=0.0040)

Supplementary Figure S4 - PFGE gel 1

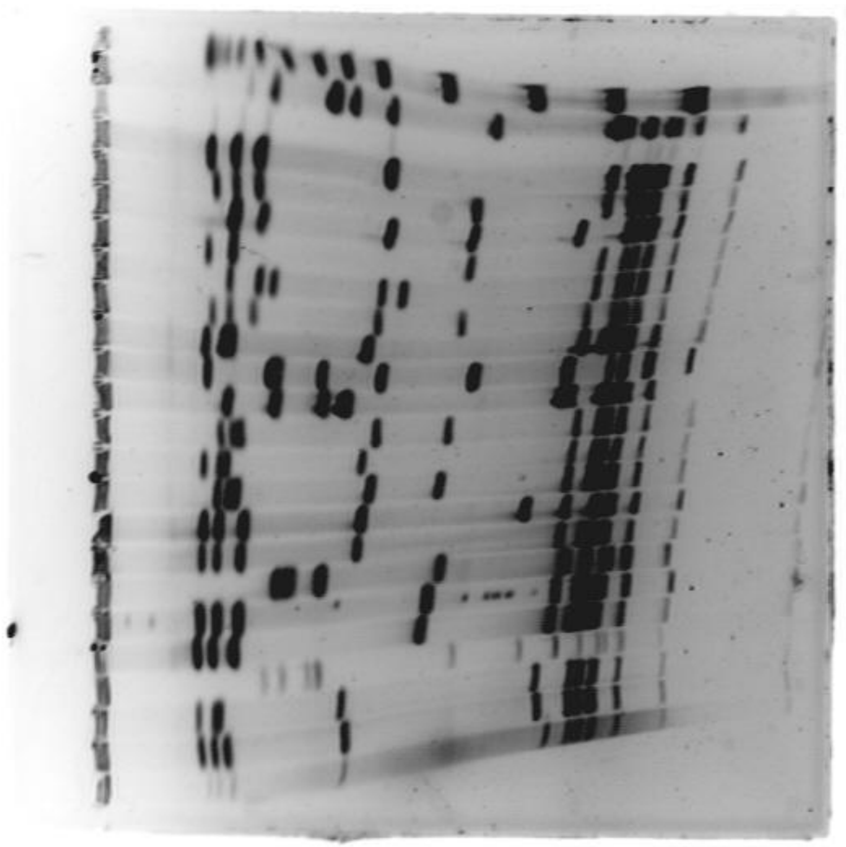

Supplementary Figure S5 - PFGE gel 2

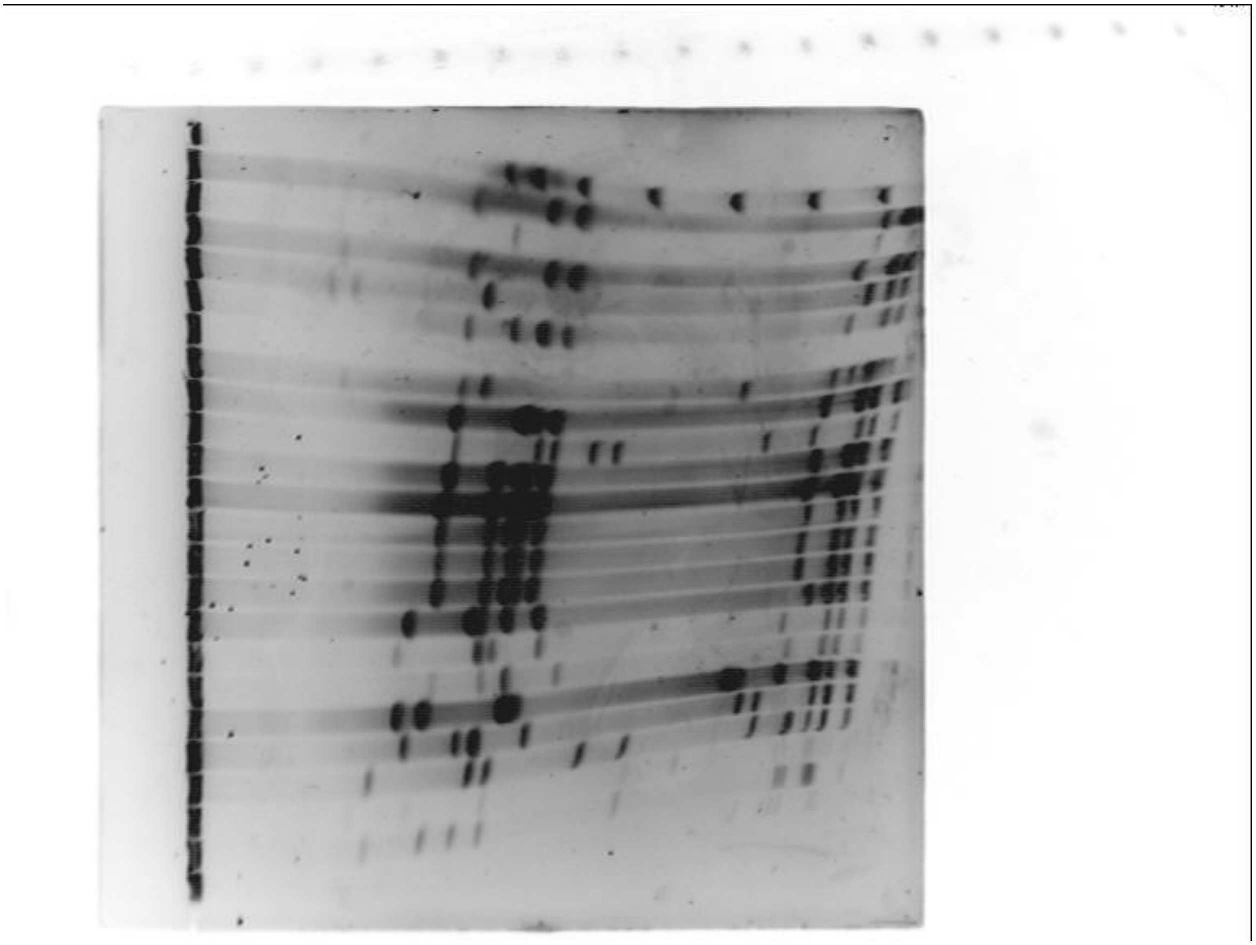

Supplementary Figure S6 - PFGE gel 3

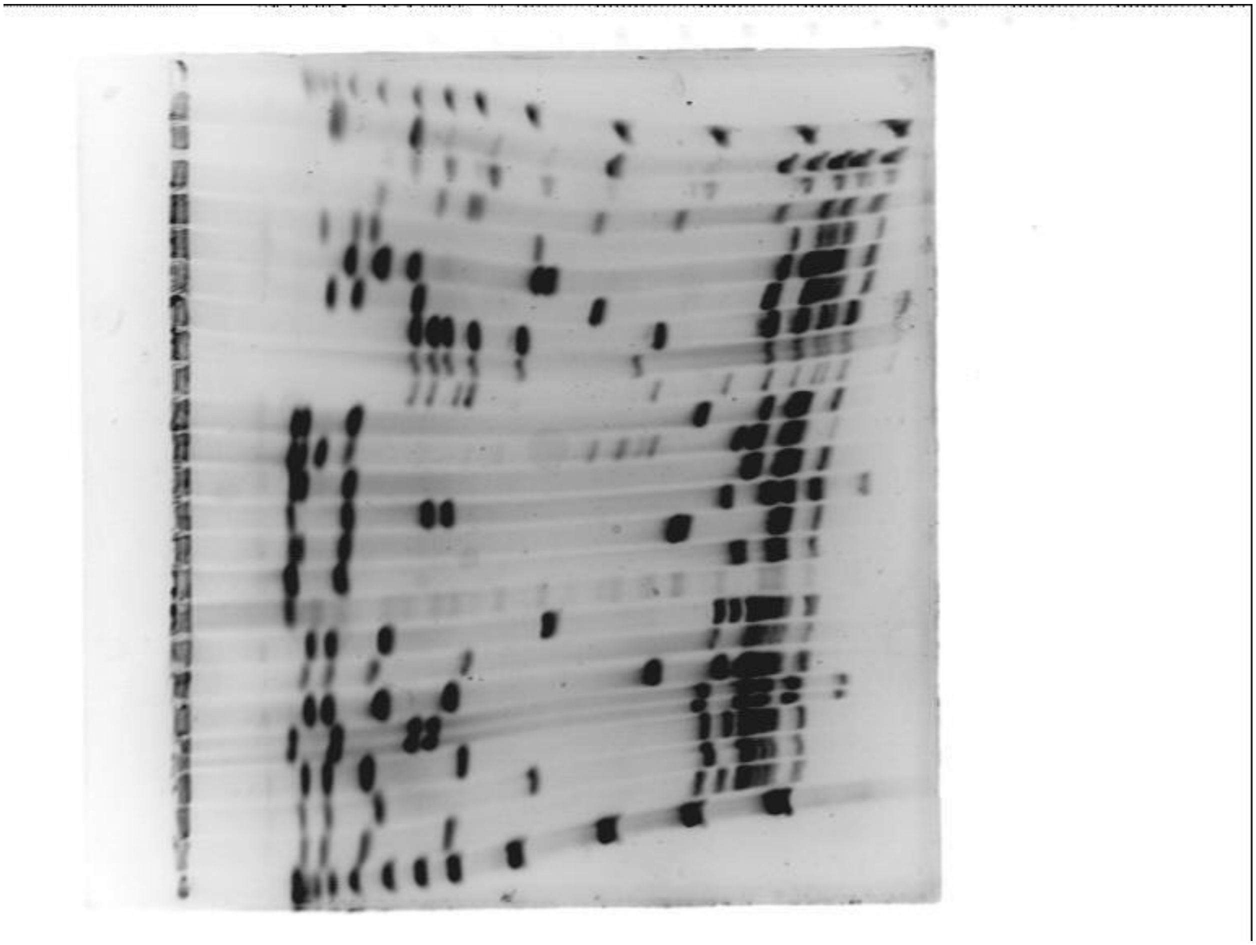

Supplementary Table 1 - The trend of the proportion of the hypervirulent clone III/ST17 within isolates of the capsular type III from 2015 to 2020.

	2015-2016	2016-2017	2017-2018	2018-2019	2019-2020	p (CA)
III/ST17 N (%)	35 (41.7)	8 (30.8)	13 (48.1)	36 (57.1)	44 (57.9)	0.0082

Supplementary Table 2 - Trends of the overall resistance rates of Group B streptococci to erythromycin, clindamycin, and tetracycline from 2015 to 2020.

	2015-2016	2016-2017	2017-2018	2018-2019	2019-2020	p (CA)
Erythromycin N (%)	56 (19.7)	30 (24.4)	35 (34.0)	76 (29.6)	89 (29.2)	0.0035
Clindamycin N (%)	49 (17.3)	26 (21.1)	29 (28.2)	59 (23.2)	74 (24.1)	0.0323
Tetracycline N (%)	233 (82.0)	103 (83.7)	89 (86.4)	218 (82.6)	270 (90.9)	0.0162

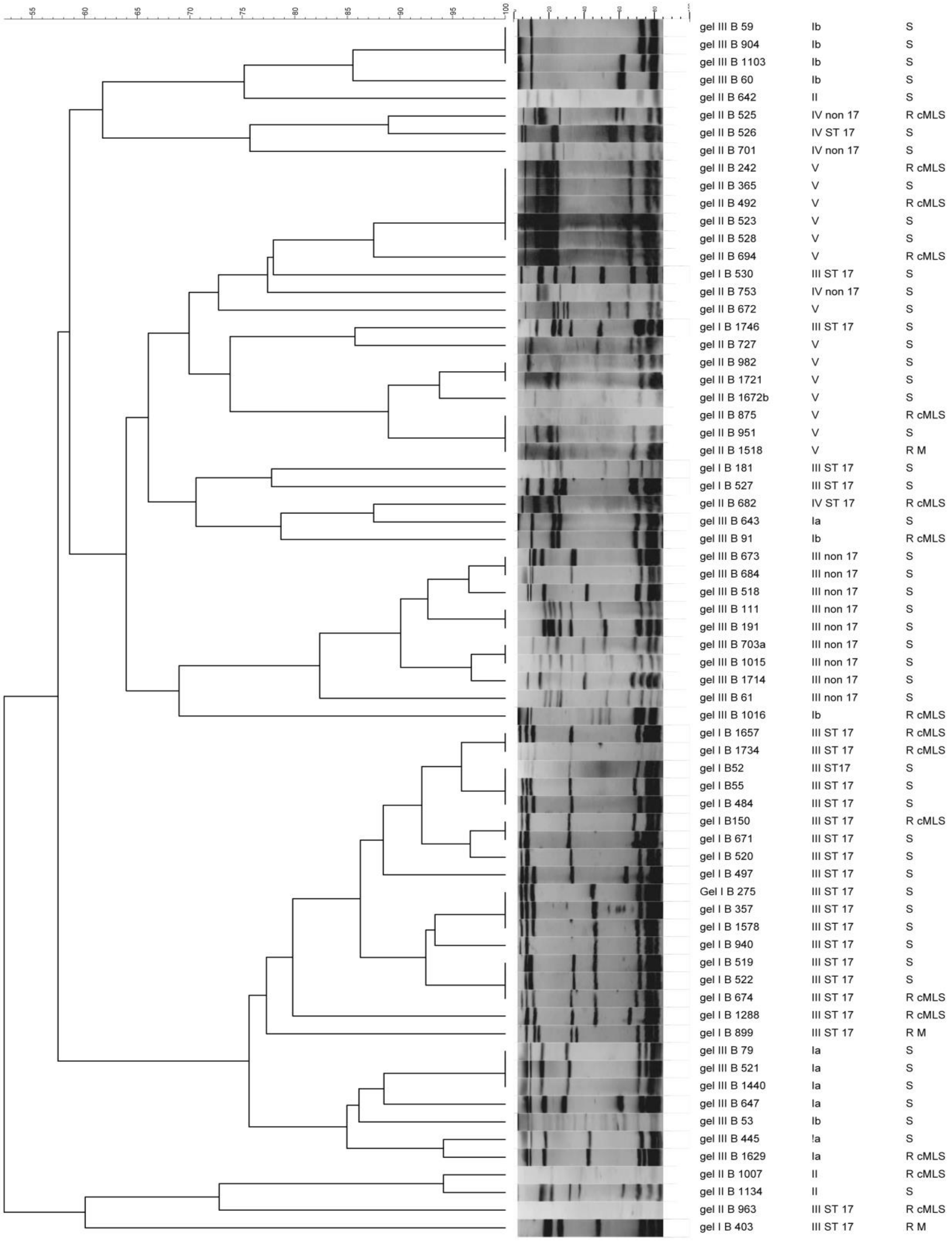

Figure 2 from the Manuscript, created by BioNumerics software. In the submitted Manuscript Figure 2 is edited for marking the 80% on the dendrogram, marking the Clusters (A-F) that we comment in the Discussion, Headers of the columns on the right, and uniforming the markings III/ST17 or non/ST17, IV/ST17 or non/ST17 like we use in the text of the Manuscript.
